# Intensive ground vegetation growth mitigates the carbon loss after forest disturbance

**DOI:** 10.1007/s11104-017-3384-9

**Published:** 2017-08-24

**Authors:** Bernhard Zehetgruber, Johannes Kobler, Thomas Dirnböck, Robert Jandl, Rupert Seidl, Andreas Schindlbacher

**Affiliations:** 1 Department of Forest Ecology, Federal Research and Training Centre for Forests, Natural Hazards and Landscape – BFW, Seckendorff-Gudent Weg 8, A-1131 Vienna, Austria; 2 Department for Ecosystem Research and Environmental Information Management, Environment Agency Austria, Spittelauer Lände 5, A-1090 Vienna, Austria; 3 Institute of Silviculture, Department of Forest and Soil Sciences, University of Natural Resources and Life Sciences (BOKU) Vienna, Peter Jordan Straße 82, 1190 Wien, Austria

**Keywords:** Disturbance, Clear-cut, Fine roots, Forest C cycling, Ground vegetation, Soil CO_2_ efflux

## Abstract

**Aims:**

Slow or failed tree regeneration after forest disturbance is increasingly observed in the central European Alps, potentially amplifying the carbon (C) loss from disturbance. We aimed at quantifying C dynamics of a poorly regenerating disturbance site with a special focus on the role of non-woody ground vegetation.

**Methods:**

Soil CO_2_ efflux, fine root biomass, ground vegetation biomass, tree increment and litter input were assessed in (i) an undisturbed section of a ~ 110 years old Norway spruce stand, (ii) in a disturbed section which was clear-cut six years ago (no tree regeneration), and (iii) in a disturbed section which was clear-cut three years ago (no tree regeneration).

**Results:**

Total soil CO_2_ efflux was similar across all stand sections (8.5 ± 0.2 to 8.9 ± 0.3 t C ha^−1^ yr.^−1^). The undisturbed forest served as atmospheric C sink (2.1 t C ha^−1^ yr.^−1^), whereas both clearings were C sources to the atmosphere. The source strength three years after disturbance (−5.5 t C ha^−1^ yr.^−1^) was almost twice as high as six years after disturbance (−2.9 t C ha^−1^ yr.^−1^), with declining heterotrophic soil respiration and the high productivity of dense graminoid ground vegetation mitigating C loss.

**Conclusions:**

C loss after disturbance decreases with time and ground vegetation growth. Dense non-woody ground vegetation cover can hamper tree regeneration but simultaneously decrease the ecosystem C loss. The role of ground vegetation should be more explicitly taken into account in forest C budgets assessing disturbance effects.

**Electronic supplementary material:**

The online version of this article (https://doi.org/10.1007/s11104-017-3384-9) contains supplementary material, which is available to authorized users.

## Introduction

Despite a growing number of silvicultural alternatives clear-cutting is still the most common harvesting practice and represents one of the primary anthropogenic disturbance regimes in many forest ecosystems (Puettmann et al. 2015). Furthermore, natural disturbance regimes such as windthrow or bark beetle infestations are common in many forest ecosystems, and may intensify under climate change (Seidl et al. 2014a). Forest management often salvages trees from these naturally disturbed sites, further enlarging their areal extent. Stand replacing disturbances therefore play a particularly important role in shaping forest landscapes. The combination of natural and anthropogenic disturbances results in forest landscapes structured by cleared areas of varying sizes, from several square meters to square kilometers (Franklin et al. 2002; Mitchell 2013; Senf et al. 2017). Consequently, stand-replacing disturbances are major drivers of the forest C cycle, and have important implications in the context of climate change mitigation (Canadell and Raupach 2008).

Forest clearings, whether of natural or anthropogenic origin, loose C to the atmosphere (Odum 1969; Körner 2003; Amiro et al. 2010; Goetz et al. 2012). C uptake by the vegetation is largely diminished after disturbance, whereas C loss from debris and the forest soil continues, or even increases when compared to pre-disturbance conditions (Knohl et al. 2002; Kurz et al. 2008; Amiro et al. 2010; Pfeifer et al. 2011). How long a disturbed forest acts as a C source depends on the severity of the disturbance, biological legacies, and the regeneration potential of the vegetation (Brown et al. 2010, 2012; Mathys et al. 2013; Seidl et al. 2014b). Furthermore, it is crucial how the decomposition of soil organic matter (SOM) is affected (Nave et al. 2010; Köster et al. 2011; Don et al. 2012). If environmental conditions become more favorable as a result of the disturbance, i.e. warmer and/or wetter, the decomposition of SOM can be enhanced and the soil CO_2_ efflux (= soil respiration; R_s_) increased (Morehouse et al. 2008; Mayer et al. 2014). The time until a cleared temperate forest returns to a net C sink is largely determined by the speed and extent of tree regeneration (Edburg et al. 2012; Hansen 2013), and typically ranges from 5 to 20 years (Romme et al. 1986; Kolari et al. 2004; Luyssaert et al. 2007; Amiro et al. 2010; Hansen 2013). However, for a number of reasons, delayed tree regeneration after disturbance can occur. In many European forests, for instance, ungulate browsing pressure is high (Reimoser and Reimoser 2010; Schodterer 2011). Therefore, the regeneration of disturbed areas can be delayed, especially if the subsequent establishment of dense ground vegetation inhibits tree regeneration (Ammer 1996; Reimoser and Gossow 1996). This imposes the risk of massive C loss from the ecosystem (Knohl et al. 2002; Mayer et al. 2014), yet at the same time prolific ground vegetation might also take up and store significant amounts of C.

We conducted a case study in a mature montane Norway spruce forest in the Austrian limestone Alps aiming at quantifying C dynamics after clear-cutting with a special emphasis on noon-woody ground vegetation. In a prior study, Kobler et al. (2015) showed that a mature Norway spruce forest in the area serves as net C sink. We hypothesized that clear-cutting turns the forest into a substantial temporal atmospheric C source as a result of ceased C uptake by trees and more favorable environmental conditions for SOM decomposition (warmer soil). We further hypothesized that C input from fast establishing non-woody ground vegetation mitigates a part of the C loss from the clear-cut areas over time.

## Materials and methods

### Site description

The study was conducted at the Austrian long-term ecosystem research and monitoring (LTER) site “Zöbelboden”, located in the National Park “Northern Limestone Alps” (N 47°50′30″, E 14°26′30″). The site is characterised by cool, humid climate with maximum precipitation in summer (mean annual temperature and precipitation 1996–2011 were 7.8 °C and 1645 mm, respectively). The snow-free period lasts from March to December. The study site is located at about 950 m a.s.l. with moderate slopes exposed in N/NW/W directions. Soil types are highly spatially variable, and consist of Lithic to Rendzic Leptosols and Chromic Cambisols with partly stagnic characteristics (WRB 2006). The underlying bedrock is dolomite.

### Study design

We used the study site’s forest monitoring plots (Hülber et al. 2008) and disturbance maps derived from aerial photographs and field surveys to locate a disturbance chronosequence which is representative as to its tree and herbaceous layer characteristics. The studied forest consisted of the following sections: (i) an undisturbed mature forest stand (hereafter MS); (ii) a six years old clearing with no tree regeneration (hereafter PD06); and (iii) a three years old clearing with no tree regeneration (hereafter PD03).

After clear-cutting in 1910, Norway spruce (*Picea abies* (L.) Karst.) was regenerated by planting to form the ~110 years old mature forest stand (~ 5 ha). The stand was located at a gently sloped, homogenous plateau-top exposed in N/NW direction. The undisturbed section (~ 3.5 ha) of the mature forest represented MS in our study. In 2007 and 2008, a section of the stand was hit by storms. Trees broken by wind were salvaged 2008, causing a cleared area of approximately ~0.5 ha. Harvest residues were left on site. Until the study year 2014, no tree regeneration had established at this clear-cut section (PPD 06), which was covered by dense grassy ground vegetation with a height of >1 m during peak growth. A further section of the stand was disturbed in 2011 following bark beetle infestation. Visibly infested and surrounding trees were felled and the stems were removed, leaving a cleared area of ~1 ha. Harvest residues were left on site as in PD06. The 2011 clearing (PD03) showed no tree regeneration and only moderate herbaceous ground vegetation cover. The direct spatial vicinity of the undisturbed stand and the two clearings ensured uniform site and soil conditions. Detailed characteristics of the different stand-sections are presented in Table 1. An areal overview of the different stand section is provided in Fig. A1.

**Table 1:** Vegetation and soil characteristics

Stage/stand-section		Mature stand (MS)	2011 clearing (PD03)	2008 clearing (PD06)
Trees				
	Tree species	Norway spruce (*Picea abies* (L.) Karsten)		
		European larch (*Larix decidua* Mill.)		
		European beech (*Fagus sylvatica* L.)		
	Basal area [m^2^ ha^−1^]	65.3		
Herb layer				
	Dominant species	*Calamagrostis varia* (Schrad.) Host.	*Cirsium arvense* (L.) Scop.	*Calamagrostis epigejos* (L.) Roth
		*Brachypodium sylvaticum* (Huds.) PB.	*Calamagrostis epigejos* (L.) Roth	*Calamagrostis varia* (Schrad.) Host.
		*Hordelymus europaeus *(L.) Jessen ex Harz	*Calamagrostis varia* (Schrad.) Host	*Carex alba* Scop.
		*Senecio ovatus* (G. Gaertn., B. Mey. & Scherb.) Willd.	*Brachypodium sylvaticum* (Huds.) PB.	*Rubus fruticosus* agg.
	Herb biomass [t C ha^−1^]	0.32 ± 0.08	1.16 ± 0.45	2.26 ± 0.65
Fine roots				
	Fine roots (0–10 cm) [t C ha^−1^]	1.56 ± 0.68	0.40 ± 0.50	0.79 ± 0.34
Soil characteristics				
	Soil organic layer (LF) thickness [cm]	0.5–2	3–4	0.5–2
	Soil mineral horizon (A) thickness [cm]	5.5–22	6–12	7–20
	C_org_ A (0–10 cm) [g kg^−1^]	94.4 ± 26.7	119.5 ± 35.8	131.7 ± 41.0

Herb biomass was harvested at six random locations within each section (mean ± SD). Fine root biomass was determined by 12 soil cores (0–10 cm soil depth) per section (mean ± SD). Soil organic C contents of the A-horzion were estimated by additional 12 soil cores (mean ± SD). Soil layer thickness was assessed at five random locations within each stand section

### Soil respiration, temperature and moisture measurements

At all stand-sections, soil CO_2_ efflux (R_s_) was measured from 12 randomly distributed plots. Each 1 × 1 m plot was equipped with a single collar for R_s_ measurements (10 cm diameter, 4 cm height, center of the plot). The PVC collars were inserted 2 cm into the ground. R_s_ was measured every three weeks from March 2014 to November 2014. R_s_ was measured with a portable infrared gas analyzer (EGM-4) and an attached chamber (SRC-1) (PP Systems International, Inc. Amesbury, MA, USA). The chamber closure time was 120 s. R_s_ was calculated automatically by fitting a quadratic function to the increasing CO_2_ headspace concentration. Soil respiration measurements of all chambers were completed within 8 h. To assure a consistent measurement protocol, the R_s_ measurements started between 9:00 and 10:00 a.m. and the collars were measured in random order.

Adjacent to the CO_2_ chamber soil temperature and soil moisture were recorded at the time of R_s_ measurement. Soil temperature was measured at 5 cm soil depth using a handheld temperature probe. Soil moisture was recorded at a soil depth of 0–15 cm using a Time Domain Reflectometry (TDR) unit (model 6050X1, Soil Moisture Equipment Corp., CA, USA) equipped with 15 cm long stainless steel rods. TDR measurements were carried out at three random locations at each of the 12 plots. Additionally, we buried between four and five permanent temperature data loggers (iButton® devices, Maxim Integrated, San Jose, CA, USA) at each stand-sections at 5 cm soil depth in Nov 2013. Using these data loggers, soil temperature was continuously recorded in an interval of three hours until Nov 2014.

To estimate the contribution of autotrophic respiration to the soil CO_2_ efflux we followed two approaches. We used already existing trenching plots (Kobler et al. 2015) to estimate the autotrophic (R_a_) and heterotrophic (R_h_) contribution to R_s_ in the mature stand. To estimate R_a_ from the dense herbaceous and grass vegetation, we established clipping plots at PD06. About 3 m away from each of the collars for periodic CO_2_ measurements, random areas of 1 m^2^ were clipped, resulting in a total of 12 clipping plots. All aboveground plant components were clipped and removed from the plots and a collar was placed in the center of each clipping plot for R_s_ measurements. Clipping was repeated one day before each CO_2_ measurement campaign. Fern mats (side length: 0.3 m, height 0.2 m) were placed in a bow above the collars to produce shade and to avoid soil heating at the clipped plots. The clipping experiment started with vegetation growth in May. The method aimed at removing all above ground vegetation components, and thus excluding the transport of newly assimilated carbohydrates to the roots. We observed that even repeated clipping did not kill the ground vegetation. Hence, a portion of the roots likely remained active, especially during the earlier stages of the clipping experiment (Högberg et al. 2001; Zhou et al. 2007). Therefore, only the difference between the soil CO_2_ efflux from untreated and clipped plots during the latter part of the study year was used as a proxy of R_a_. Due to limited labour resources, we did not clip at PD03. For PD03, we estimated the autotrophic contribution by using the relationship between fine root carbon (*f* [t C ha^−1^ yr.^−1^]) and the estimated autotrophic soil CO_2_ efflux (*a* [%]) from PD06:

1 $$ PD03\ a=\frac{PD06\ a}{PD06\ f}\times PD03\ f $$

### Litter and ground vegetation biomass sampling

Five litter collectors (0.68 m diameter) were placed in MS in November 2013. Litter collectors were emptied in March and November 2014. The litter was oven-dried at 105 °C and weighed. The C content of litter was assumed as 50% of the dry weight (De Wit et al. 2006).

To derive an estimate of litter input from ground vegetation, ground vegetation was harvested at the end of the vegetation period in September 2014 from all sites. On each site, six random plots were selected. The above ground vegetation was clipped inside a wooden frame (0.5 × 0.5 m), oven-dried at 105 °C and weighed. The C content of biomass was assumed as 47.5% of the dry weight (Schlesinger 1991). We assumed that the harvested ground vegetation biomass roughly resembled the annual (above) ground vegetation litter input.

### Fine root biomass, soil C and N and microbial biomass

At each of the 12 plots per section, a soil core (7 cm diameter) for fine root analyses was taken in the vicinity of the CO_2_ collar, in June 2014. Coring depth was 10 cm, as most of the fine roots were considered in the upper soil layer (> 80% of total fine root biomass was found at 0–10 cm soil depth at both the disturbed and undisturbed study locations; Kobler unpublished data). Fine roots were washed to remove all soil particles. Root fragments were picked out of the samples with tweezers, sorted into living and dead, and further ordered by diameter and origin (grass roots, tree roots). The roots were considered as living when the stele was bright and resilient (Vogt and Persson 1991). Roots less than 2 mm in diameter were classified as fine roots. After sorting, fine roots were oven-dried at 105 °C. The C content of roots was considered to be 50% of the dry weight (De Wit et al. 2006).

A second soil core per plot was taken to assess organic C and N contents of the upper 10 cm mineral soil. Total C and N contents of the soil horizons were determined with a LECO CN-2000 dry combustion analyzer (LECO Corporation, MI, USA). Organic C content was assessed by correcting total soil C by carbonate contents (ISO 10694; www.iso.ch). Microbial biomass C and N were determined using a modified version of the chloroform fumigation extraction (CFE) method (Schinner et al. 1996). 10 g of homogenized soil were weighed into 100 ml Erlenmeyer flasks to be chloroform fumigated and 5 g were weighed into plastic beakers as control samples. The soil samples in the Erlenmeyer flasks were kept inside a desiccator with sodium lime and wet filter papers within a chloroform atmosphere for 24 h at 25 °C. After fumigation the samples were split into two 5 g samples. 25 ml of 2 M KCl solution were added to the samples that were then shaken for 30 min and afterwards filtered through N-free filters. Control samples were processed using the same procedure. The C and N content of the KCl extracts were measured with a TOC-V CPH E200V soluble analyzer linked with a TN-unit TNM-1220 V (Shimadzu, Kyoto, Japan). For calibration a dilution series of a standard stock solution was added. Microbial biomass C and N contents in μg g^−1^ dry matter were calculated by subtracting the C and N contents of the control sample from the mean C and N contents of the two fumigated samples.

### C budgeting

In a first step, we used an exponential model (Eq. 2) between soil temperature and soil respiration to estimate the annual soil CO_2_ efflux of each individual plot (Janssens et al. 2003).

2 $$ {R}_s={F}_{10}\ {Q_{10}}^{\left(\frac{T-10}{10}\right)} $$

*R*_*s*_ was the soil CO_2_ efflux rate (μmol CO_2_ m^−2^ s ^−1^), *T* the soil temperature (°C) at a soil depth of 5 cm, *F*_*10*_ the soil CO_2_ efflux at a soil temperature of 10 °C and *Q*_*10*_ denoted the factor by which R_s_ increases when the soil temperature is rising by 10 °C. Nonlinear modelling was performed by means of the R package “minpack.lm” (Elzhov et al. 2013).

Equation (2) was parameterized for each individual plot including the clipping plots, as well as for the trenching and control plots, using the observed R_s_ in combination with the manually measured soil temperature at a depth of 5 cm. Subsequently, daily and annual plot specific cumulative R_s_ were calculated from the models using the high temporal resolution soil temperature data.

The basal area, mean tree height, and stem volume of the mature stand were determined by angle-count sampling (5 angle-count samples per stand) (Bitterlich 1984). We used a detailed assessment of biomass stocks and increment of the stem, branch and coarse root compartments of a comparable adjacent stand with similar tree species composition, tree age, and management history (Kobler et al. 2015) to deduce the standing biomass and biomass increment of the mature stand. Biomass stocks and increment of the mature stand were adjusted by the observed difference in stem volume between the two neighbouring stands. Net ecosystem productivity (NEP) was calculated as:

3 $$ \mathrm{NEP}=\mathrm{annual}\  \mathrm{biomass}\  \mathrm{increment}+\mathrm{annual}\  \mathrm{litterfall}-\mathrm{annual}\ {\mathrm{R}}_{\mathrm{h}} $$

For the two clearing sites (PD06, PD03), we assumed that the entire aboveground biomass stock of the ground vegetation annually enters the soil as litter. Therefore, the NEP was calculated as litter input minus R_h_. Turnover rate for fine roots was considered as 0.8 yr.^−1^ (Gill and Jackson 2000) for trees and ground vegetation.

### Statistical analysis

Effects of time since disturbance on soil CO_2_ efflux, soil temperature, and soil moisture were assessed by means of repeated measures ANOVA and post hoc pairwise student t tests with Bonferroni *p*-value adjustment. Effects of time since disturbance on accumulated R_s_, soil C and N contents, ground vegetation biomass, fine root biomass and microbial biomass were analysed by means of one-way ANOVA. In the case of significant effects (*p* < 0.05), post-hoc comparisons were made using Tukey’s honest significant difference (HSD) test. We used square root or logarithmic transformations to meet the assumptions of normality and homogeneity of variance. We used Pearson’s correlation coefficient to analyse the relationship between the annual R_s_ and the microbial biomass respectively the soil organic C as well as the relationship between fine root biomass and soil organic C and N. All statistics were carried out with R (R Core Team 2015) at a significance level of 95%.

## Results

### Soil temperature, moisture and respiration (R_s_)

Average soil temperatures during R_s_ measurements were 9.6 ± 0.3 °C (MS), 10.1 ± 0.4 °C (PD06) and 10.6 ± 0.4 °C (PD03). Mean annual (continuously measured) soil temperatures were 8.2 ± 0.2 °C (MS), 8.0 ± 0.3 °C (PD06) and 8.5 ± 0.3 °C (PD03). Differences between mean annual soil temperatures as well as soil temperatures during R_s_ measurements did not differ significantly between MS and the clear-cut areas. Mean soil moisture was 39.5 ± 0.7% (MS), 49.2 ± 0.9% (PD06) and 52.3 ± 0.9% (PD03). Mean soil moisture contents differed significantly between the forest sections (*p* = 0.028). Post hoc test showed that soil moisture was different between all three sections (*p* < 0.022).

**Fig. 1:**
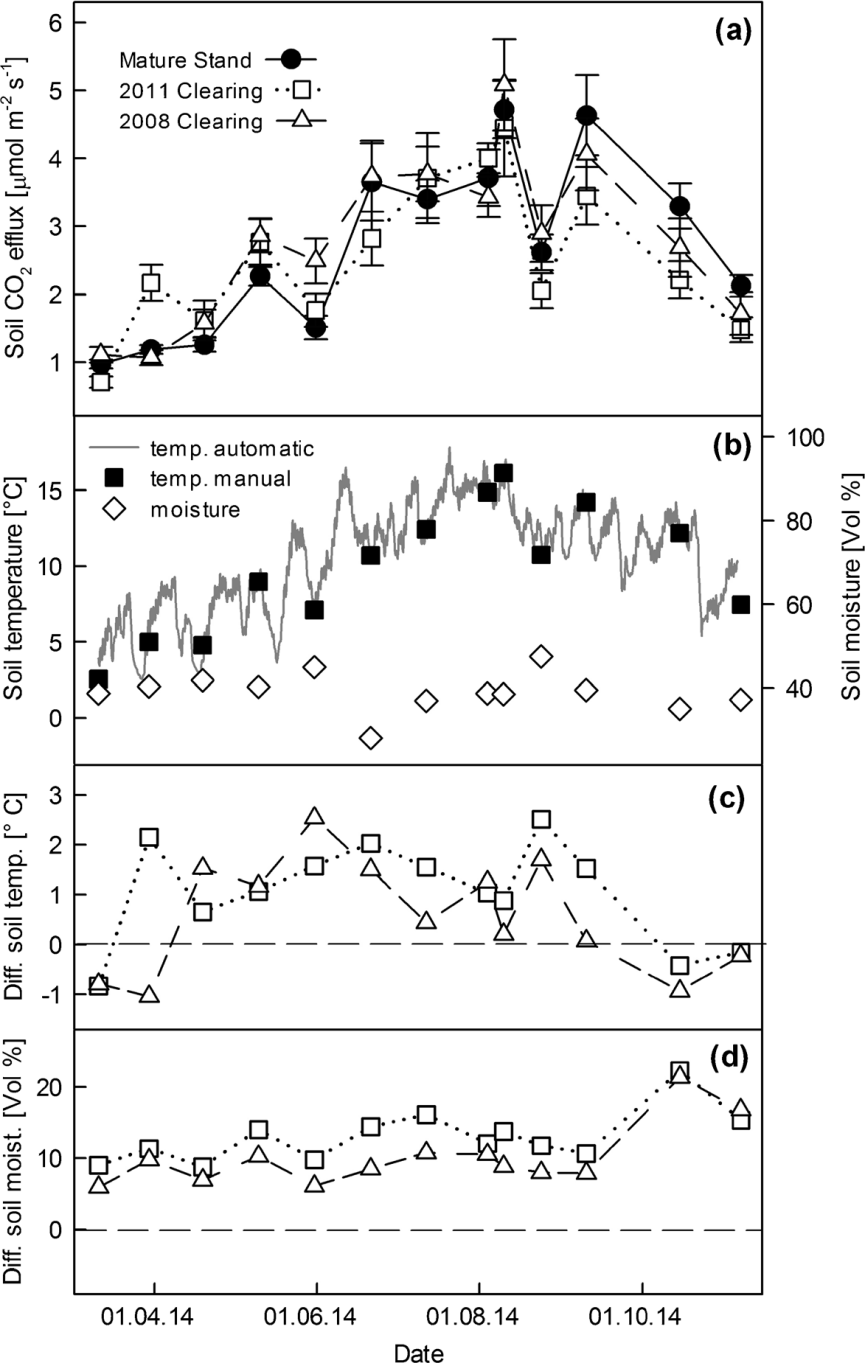
**a** Soil CO_2_ efflux of the different stand sections (mean ± SE, *n* = 12). **b** Manually measured and high resolution soil temperature and soil moisture of the mature stand (MS). Deviations of soil temperature (**c**) and moisture (**d**) from that of the mature stand (MS = dashed zero line). Open squares represent the more recently disturbed stand section (PD03, 2011 clearing) and open triangles represent the less recently disturbed stand section (PD06, 2008 clearing)

R_s_ exhibited a clear seasonal pattern following changes in soil temperature, with the peak respiration rates recorded during summer (Fig. 1a). Mean measured R_s_ over the study period was 2.60 ± 0.13 μmol CO_2_ m^−2^ s ^−1^ (MS), 2.73 ± 0.14 μmol CO_2_ m^−2^ s ^−1^ (PD06) and 2.45 ± 0.13 μmol CO_2_ m^−2^ s ^−1^ (PD03). Mean measured R_s_ did not differ significantly between stand sections. The temperature driven R_s_ model (Eq. 2) explained between 79 and 86% of the temporal variation in measured R_s_. The model slightly overestimated R_s_ at lower R_s_ rates and slightly overestimated R_s_ at higher R_s_ rates (Fig. A2). The modelled annual R_s_ was 8.88 ± 0.28 t C ha^−1^ (MS), 8.85 ± 0.28 t C ha^−1^ (PD06) and 8.53 ± 0.24 t C ha^−1^ (PD03) and did not differ significantly between stand sections.

**Fig. 2:**
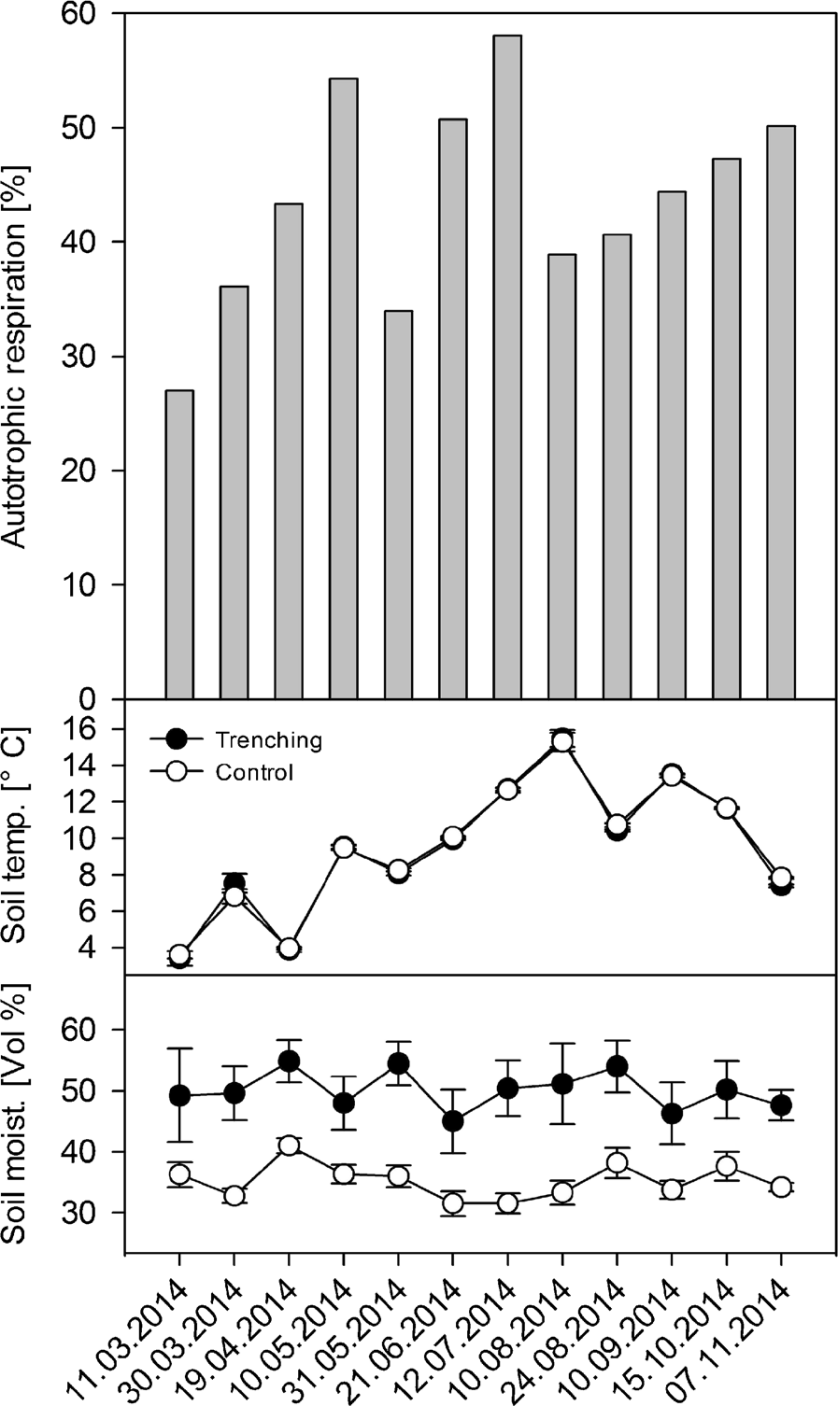
Trenching experiment at the mature stand. The upper panel shows the autotrophic contribution to R_s_. The middle and lower panels show soil temperature (mean ± SE, *n* = 12) and soil moisture (mean ± SE, *n* = 12) from the trenched and associated control plots

R_s_ of trenched plots on MS was on average 44 ± 3% lower than at the corresponding control plots (Fig. 2, Table 2). Average soil moisture was 15 ± 1 Vol% higher at trenched plots (*p* < 0.001) than at control plots (average moisture: 35 ± 1 Vol%). Mean soil temperatures were 9.5 ± 1.1 °C (trenched) and 9.5 ± 1.0 °C (control).

**Table 2:** Carbon fluxes and net ecosystem productivity (NEP) during 2014

Stage/stand-section		Mature stand (MS)		2011 clearing (PD03)		2008 clearing (PD06)	
Annual biomass increment	[t C ha^−1^ yr.^−1^]	3.8					
Soil respiration (Rs)	[t C ha^−1^ yr.^−1^]	8.9	± 0.3	8.5	± 0.2	8.9	± 0.3
Autotrophic	[%]	44		18		35	
	[t C ha^−1^ yr.^−1^]	3.9		1.5		3.1	
Heterotrophic	[%]	56		82		65	
	[t C ha^−1^ yr.^−1^]	5.0		7.0		5.8	
Tree litter	[t C ha^−1^ yr.^−1^]	1.8					
Herb litter	[t C ha^−1^ yr.^−1^]	0.3	± 0.1	1.2	± 0.5	2.3	± 0.7
Root litter	[t C ha^−1^ yr.^−1^]	1.2	± 0.5	0.3	± 0.4	0.6	± 0.3
NEP	[t C ha^−1^ yr.^−1^]	2.1		−5.5		−2.9	

Annual biomass increment represents the estimated total tree biomass increment of MS (mean ± SD). All herbal biomass (increment) was assumed to annually enter the soil (herb biomass = herb litter). Annual soil respiration was modelled for each plot individually (mean ± SD). Autotrophic and heterotrophic components were estimated by means of trenching (MS) and clipping (clearings) and annual sums were modelled for each plot and component, respectively. Tree litterfall represents a bulk sample of 5 collectors. Fine root litterfall was estimated from fine root biomass (0–10 cm depth) with a turnover rate of 0.8 yr.^−1^ (mean ± SD)

**Fig. 3:**
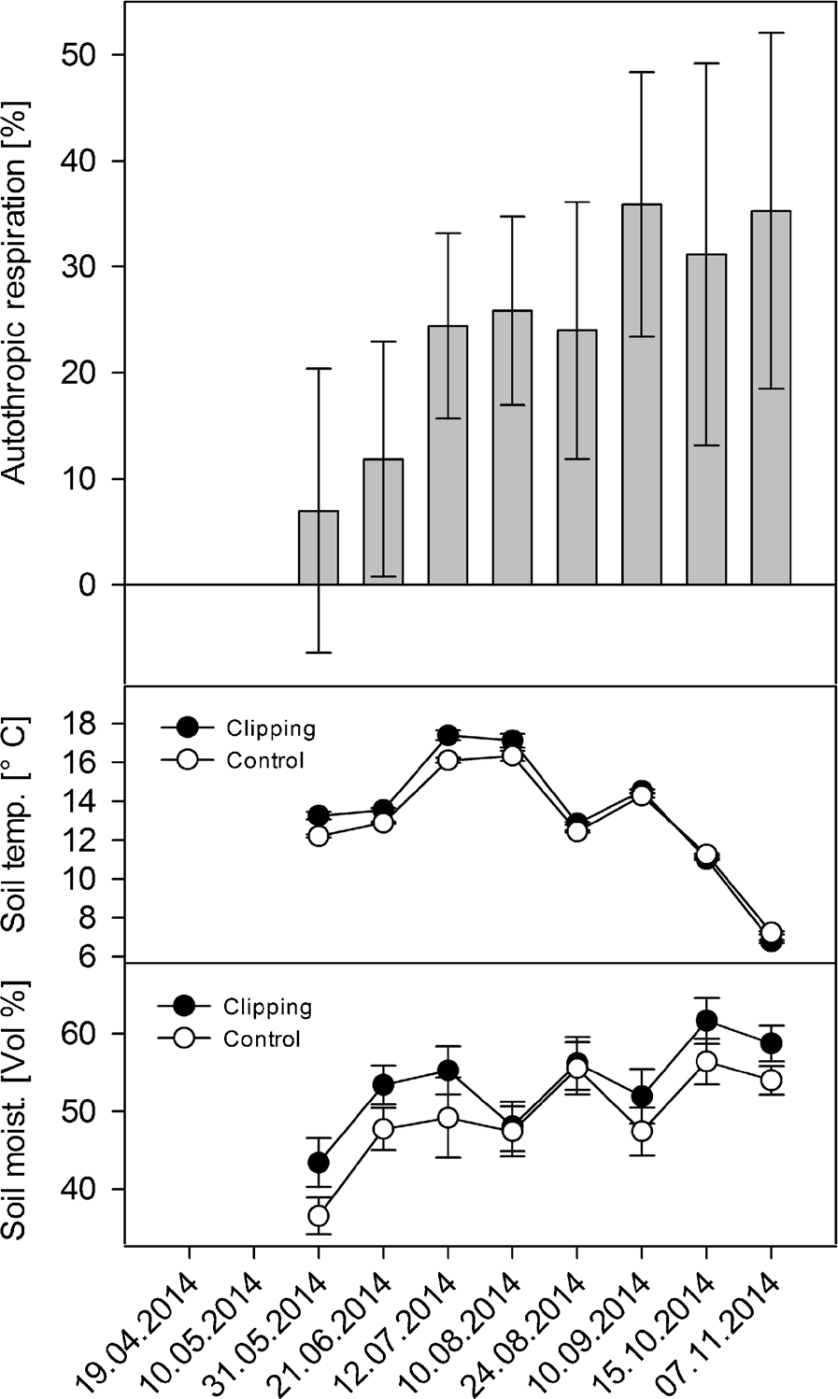
Clipping experiment. The upper panel shows the autotrophic contribution to R_s_ (mean ± SE, *n* = 12). The mid and lower panel show soil temperature (mean ± SE, *n* = 12) and soil moisture (mean ± SE, *n* = 12) from the clipping and associated control plots

There was no clear effect on R_s_ during the initial phase of clipping. After a gradual decrease, clipped plots stabilized in autumn 2014 at R_s_ rates ~35% lower than at untreated plots (Fig. 3, Table 2). Soil moisture and temperature did not differ significantly between the clipped and untreated plots.

### Litterfall and ground vegetation biomass

Aboveground tree litterfall was 1.80 t C ha^−1^ yr.^−1^ and ground vegetation biomass was 0.32 ± 0.08 t C ha^−1^ at MS (Tables 1 and 2). Harvested ground vegetation biomass amounted to 2.26 ± 0.65 t C ha^−1^ yr.^−1^ at PD06, and 1.16 ± 0.45 t C ha^−1^ yr.^−1^ at PD03. Differences in harvested ground vegetation biomass were statistically significant between all stand sections (*p* < 0.006).

### Soil properties, C and N contents, fine root and microbial biomass

Soil organic layer thickness varied between 0.5 and 2 cm at MS and PD06, and between 3 and 4 cm at the recent clear-cut section PD03. Mineral A-horizon thickness showed high spatial heterogeneity and ranged from 5.5–22 cm (Table 1). Soil organic C contents ranged between 94 ± 8 g kg^−1^ DW (MS) and 131 ± 11 g kg^−1^ DW (PD06) (Table 1). Soil C (*p* = 0.048) and N (*p* = 0.014) contents were significantly lower at MS when compared to PD06.

Living fine root biomass was highest at MS (1.56 ± 0.68 t C ha^−1^). The clearings showed significantly lower (*p* < 0.002) fine root biomass, but PD06 (0.79 ± 0.34 t C ha^−1^) showed nearly twice the amount of fine root biomass as PD03 (0.40 ± 0.50 t C ha^−1^, Table 1), thereby almost exactly making up the difference in aboveground biomass of PD06 and PD03 (Table 1). All living fine roots at the clearings were of ground vegetation origin, whereas tree fine roots represented ~90% at MS.

Microbial biomass C amounted to 0.76 ± 0.26 mg g^−1^ DM (MS), 0.61 ± 0.40 mg g^−1^ DM (PD03) and 0.60 ± 0.18 mg g^−1^ DM (PD06). Due to the high spatial heterogeneity the differences were not statistically significant. The cumulative annual R_s_ of the individual stages/stand-sections neither correlated significantly with microbial biomass C, soil organic C and N contents, nor with fine root biomass.

### C budgeting

Estimated NEP and its components are presented in (Table 2). MS was a C sink to the atmosphere, while PD06 and PD03 were C sources. The estimated C loss from PD03 was nearly twice as high as the estimated C loss from the densely vegetated PD06.

## Discussion

As hypothesized, the two disturbed sites were C sources while the mature forest stand was a C sink to the atmosphere. Although there was no tree regeneration, the C source strength of the clear-cut sites decreased significantly with time since disturbance. The C loss six years after clear-cutting was almost 50% lower than three years after clear-cutting. As hypothesized, lower heterotrophic soil respiration rates together with the high C input from the dense grassy ground vegetation decreased the ecosystem C loss with time since disturbance.

The estimated NEPs of our study are in the range of values from Kowalski et al. (2004), reporting forest stands as C sinks ranging from ca. 1.00 to 5.00 t C ha^−1^ yr.^−1^ and cleared sites as C sources of similar magnitude. Likewise, other studies report considerable C loss to the atmosphere shortly after disturbance (Knohl et al. 2002; Brown et al. 2010; Amiro et al. 2010; Edburg et al. 2012; Mathys et al. 2013). Many of these studies focused on the first 1–3 years after disturbance or on even shorter timescales during which the disturbed sites generally are strong C sources. In most longer-term studies, clearings were re-planted or regenerated naturally within a comparatively short timeframe (Kowalski et al. 2004; Humphreys et al. 2005; Takagi et al. 2009; Amiro et al. 2010; Williams et al. 2014). There are thus only a few studies which specifically address the C dynamics of clearings with slow or failed tree regeneration due to heavy competition from ground vegetation. Kolari et al. (2004), for instance, found that the NEP of a 4 year old Scots pine clearing without any tree regeneration was −2.6 t C ha^−1^ yr.^−1^, which is close to the NEP estimate of the six years old clearing (PD06) in our study (−2.9 t C ha^−1^ yr.^−1^). Studying the effects of large-scale windthrow in a similar forest ecosystem as studied here, Mayer et al. (2014, 2017) found that windthrow substantially increased soil temperatures at south facing slopes, facilitating SOM decomposition, R_s_, and R_h_. As a result, windthrown areas suffered a significant loss of soil C throughout the first six years after disturbance (Mayer et al. 2017). In contrast to our site, ground vegetation cover remained scarce even after 6 years post windthrow, and the contribution of R_a_ remained low (< 20%). Conversely, higher R_s_ rates than in our study (11 t C ha^−1^ yr.^−1^) were reported for another windthrow area 12 years after disturbance (Mayer et al. 2014). At this site, ground vegetation was comparable in density to our PD06 site. However, autotrophic and heterotrophic sources were not distinguished and the net C loss from this site thus remains unresolved.

Effects of forest disturbance on R_s_ are diverse. On the one hand, disturbance decreases or even ceases R_a_ and thereby reduces R_s_ (Zerva and Mencuccini 2005; Edburg et al. 2011; Moore et al. 2013). On the other hand, disturbance can favor the soil microclimate (i.e. increase soil temperature and/or moisture) for SOM decomposition, and additional above and below ground litter becomes available for decomposers. Therefore, post-disturbance R_s_ was frequently observed to attain levels equivalent to undisturbed forest stands (Kolari et al. 2004; Morehouse et al. 2008; Forrester et al. 2013; Mayer et al. 2014, 2017). A key factor influencing R_s_ is soil temperature. In contrast to our hypothesis, disturbance had only a minor effect on soil temperature at our site. At both clearings soil temperature was slightly elevated during most of the growing season (Fig. 1c), but the mean annual soil temperatures across the different stand-sections were similar. The reason was that soils at the clearings remained cooler during spring, as snow cover disappeared several weeks later compared to the stand with closed canopy (Fig. A3). The positive influence of direct sunlight on soil temperature was limited by the NW exposition and the flat terrain of our site, e.g. compared to the S exposed slopes studied by Mayer et al. (2014, 2017). In our study, parts of the clearings had been affected by shading of the neighbouring mature trees, especially during seasons with lower solar altitude. Shading had been found to significantly affect soil temperature and Rs in forest gaps (Schatz et al. 2012). The comparably small size (0.5–1 ha) of the investigated clearings could therefore be a further reason that only minor changes in soil temperature were observed. In contrast to soil temperature, soil moisture was significantly higher in the clearings. This is a frequently observed pattern, attributable to the lack of water uptake and evapotranspiration by trees (Palviainen et al. 2004). The higher water availability likely had only minor effects on R_s_ because soil moisture never became a limiting factor to decomposer microbes at any disturbed or undisturbed stand section (Fig. 1b).

If temperature and moisture effects on R_s_ were minor, then other factors must have attained R_s_ at the two clearings. The organic layer, which mainly consisted of decomposing needles and twig compartments, was significantly thicker at the recent clearing (PD03) (Table 1). This indicates that easily decomposable organic material from tree residues was still readily available to decomposers at PD03, and that its decomposition contributed to the soil CO_2_ efflux. The same likely holds true for below ground fluxes, where dead roots of the recently killed trees were still available for decomposition. Lower absolute R_h_ at PD06 (Table 2) also points towards a decline in readily available labile SOM/litter over time since disturbance.

A significant contribution of R_s_ was autotrophic at PD06, i.e., the site which was covered by dense predominantly grassy ground vegetation. Our estimated 35% contribution of R_a_ is in line with results from other clipping experiments in grasslands (20–50%) (Kuzyakov and Cheng 2001; Wan and Luo 2003; Bahn et al. 2006), and with that of a trenching experiment at a clear-cut site in Harvard forest (34%) (Williams et al. 2014). We repeatedly clipped with the intention to kill the grassy ground vegetation and to offset root respiration. However, even after a whole growing season of repeated clipping, grasses were still alive and resprouted in-between consecutive R_s_ measurements. Therefore, root respiration still contributed to R_s_ at our clipping plots. The 35% R_a_ is thus a conservative estimate, and the real autotrophic contribution at PD06 was likely higher (and the soil C losses to the atmosphere correspondingly lower). Annual estimated R_s_ from MS falls within values from other temperate forests (e.g. Knohl et al. 2008; Schindlbacher et al. 2014; Mayer et al. 2014). The contribution of R_a_ to R_s_ (44%) was within values of comparable mature forest ecosystems as well (Hanson et al. 2000; Zerva and Mencuccini 2005; Subke et al. 2006).

Similar to the clipping method, trenching holds uncertainties. Trenched plots, for instance, had slightly higher soil moisture contents as control plots. Differences in soil moisture can cause methodological bias if control plots dry out during certain drought periods, or if SOM decomposition becomes unfavorable when trenched plots are fully water saturated (Díaz-Pinés et al. 2010). In our study, moisture-induced bias was unlikely because soil moisture content in control and trenched plots never became limiting or oversaturated (Fig. 2). Further uncertainty arises from decomposition of dead roots in trenching plots, which can add to the soil CO_2_ efflux. Since the trenching plots were already established 5 years prior to our study (Kobler et al. 2015), most dead fine roots should, however, have been decomposed by the time our measurements took place. Nonetheless, due to the number of caveats, trenching provides only a rough proxy of autotrophic and heterotrophic contributions to overall respiration. R_h_ is the major C outflux of the ecosystem, and uncertainty in its contribution will have considerable impacts on the C budget. A > 80% heterotrophic contribution to R_s_ would turn MS to a C source. Such a huge deviation of our estimate (66%), however, is far beyond the expected uncertainty related to the trenching method.

A further pathway of C loss is leaching of dissolved organic carbon (DOC) or dissolved inorganic carbon (DIC) of biogenic origin (Kindler et al. 2011; Schindlbacher et al. 2015). In our study, we did not assess C leaching. The magnitude of C loss via leaching is several times lower than that via R_h_. Schindlbacher et al. (2009), for instance, estimated DOC leaching of 1.5–3 g m^−2^ yr.^−1^ for a mature spruce stand at a similar site, which falls well within the range of values for other temperate forests in Europe (Kindler et al. 2011). It is likely that PD03 and PD06 showed higher C leaching when compared to MS, as overall seepage likely was higher on the cleared sites (Neff and Asner 2001). However, due to the minor contribution of C leaching to the overall soil C loss, potential clearing effects on C leaching can be expected to add only insignificantly to our NEP estimates.

To come up with a realistic soil C budget, it is not only important to assess the C loss but also the C input to the system. Annual above-ground tree litter estimates were robust and fall within values from other temperate mountainous forests (Perruchoud et al. 1999; Caprez et al. 2012). The dense grassy ground vegetation at the six years old clearing (PD06) was dominated by *Calamagrostis epigejos* and other graminoids (Table 1). These communities are common and widely distributed in Central European forests, especially where the natural regeneration of trees is limited (Máliš et al. 2013). Above-ground living biomass at PD06 was 2.3 t C ha^−1^, which is in the range of similar plant communities reported elsewhere (1.5 t C ha^−1^ (Pysek 1991), 3.1 t C ha^−1^ (Máliš et al. 2013)). At PD03, ground vegetation biomass was lower (1.2 t C ha^−1^), which shows that the improved light availability promotes a rapid spread of herbaceous plants and grasses in cleared areas (Ammer 1996; Palviainen et al. 2005; Donoso and Nyland 2006; Naaf and Wulf 2007; Kern et al. 2012; Kramer et al. 2014; Pröll et al. 2015). In our study, we used the above ground biomass as a proxy for the annual above ground litter input. The true above ground litter input might have been higher, as the ground vegetation biomass was sampled in September, and further biomass growth thereafter is likely. Moreover, potential above ground litter input prior to biomass harvesting was not accounted for.

Our tree fine root biomass estimates (1.6 t C ha^−1^) are within the range of those observed for similar Norway spruce forests (Helmisaari et al. 2007; Brunner et al. 2013). The fine root biomass from the dense ground vegetation at PD06 (0.8 t C ha^−1^) falls within fine root biomass estimates at grassland sites (Solly et al. 2013). The turnover rate (0.8 yr.^−1^) used to calculate the below ground C input via fine roots is within the average of reported values (Finér et al. 2011; Brunner et al. 2012). It, however, needs to be noted that only the top 10 cm of the soil were sampled and analyzed for fine root biomass in our study. Although most Norway spruce fine roots are typically located in the top-soil (Ostonen et al. 2005), a fraction of fine roots is likely present also in soil layers deeper than 10 cm. Accordingly, our estimate for tree fine root biomass and corresponding litter input at MS is a conservative estimate. At the two forest clearings, the 10 cm root coring should have reached most of the rooting zone of the ground vegetation species (Ostonen et al. 2005; Wu et al. 2011). The biomass increment estimate of MS is likely to be robust, as it was derived from a detailed assessment of a close-by stand (200 m away) of the same age, stand and site properties (Kobler et al. 2015).

Notwithstanding all limitations and uncertainties, we are confident that the applied methods were adequate to answer our prime question, how non-woody ground vegetation growth affected the C balance of poorly regenerating forest clearings. To avoid over-interpreting the effects of ground vegetation on C cycling, we kept our estimates of ground vegetation, autotrophic soil respiration, litter input and fine root turnover conservative (see above). Therefore, the estimated C losses from forest clearings presented here are an upper bound of the potential C loss. Spatial replication among various disturbance sites/chronosequences could further illustrate whether the results of our case study apply at larger spatial scales.

Our results indicate that fast growing grassy ground vegetation can mitigate parts of the ecosystem C loss after disturbance. However, from a forest management and climate change mitigation perspective, un-stocked sites such as the ones studied here, remain largely un-desirable. Once established, dense grassy ground vegetation strongly inhibits tree regeneration (Pröll et al. 2015). Consequently, tree planting and tending often remains the only pathway towards successfully establishing the next generation of trees under these conditions. Such measures are labour intensive and costly, and could be avoided by reducing inhibiting factors such as browsing pressure, before a dense grass cover takes over. On the other hand, patchy and open habitats have considerable value for biodiversity, as they provide habitat for a wide range of species groups (Swanson et al. 2011; Thom et al. 2017).

## Conclusions

Here we show that disturbed areas in a central European mountain forest initially lose high amounts of C to the atmosphere. Our analyses highlights that while tree regeneration is slow or absent, the establishment of a cover of fast-growing non-woody ground vegetation, together with decreasing heterotrophic soil respiration, can reduce the ecosystem C loss in the first years after disturbance. Although largely undesirable from a forest management perspective, slowly regenerating forest clearings loose less C than suggested from short term studies extrapolating fluxes from the initial years after disturbance, and ignoring the role of ground vegetation in the forest C cycle.

## Electronic supplementary material

ESM 1 (DOCX 563 kb)
