# Supplementary material for: Intensive ground vegetation growth mitigates the carbon loss after forest disturbance
Source: Plant Soil. 2017 Aug 24;420(1):239–52. doi: 10.1007/s11104-017-3384-9 (PMC5711974; doi:10.1007/s11104-017-3384-9)
Supplement: Supplementary file 1 — (DOCX 563 kb) [file 11104_2017_3384_MOESM1_ESM.docx]

**Supplementary Figures**


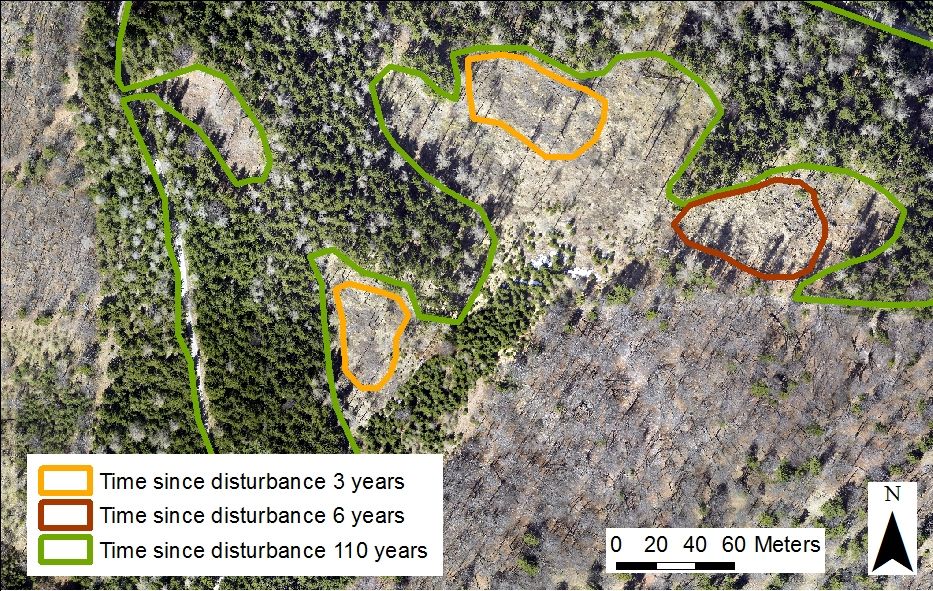


**Figure A1:** Overview map of the study sites: time since disturbance of 3 (PD03), 6 (PD06) and 110 years (MS).


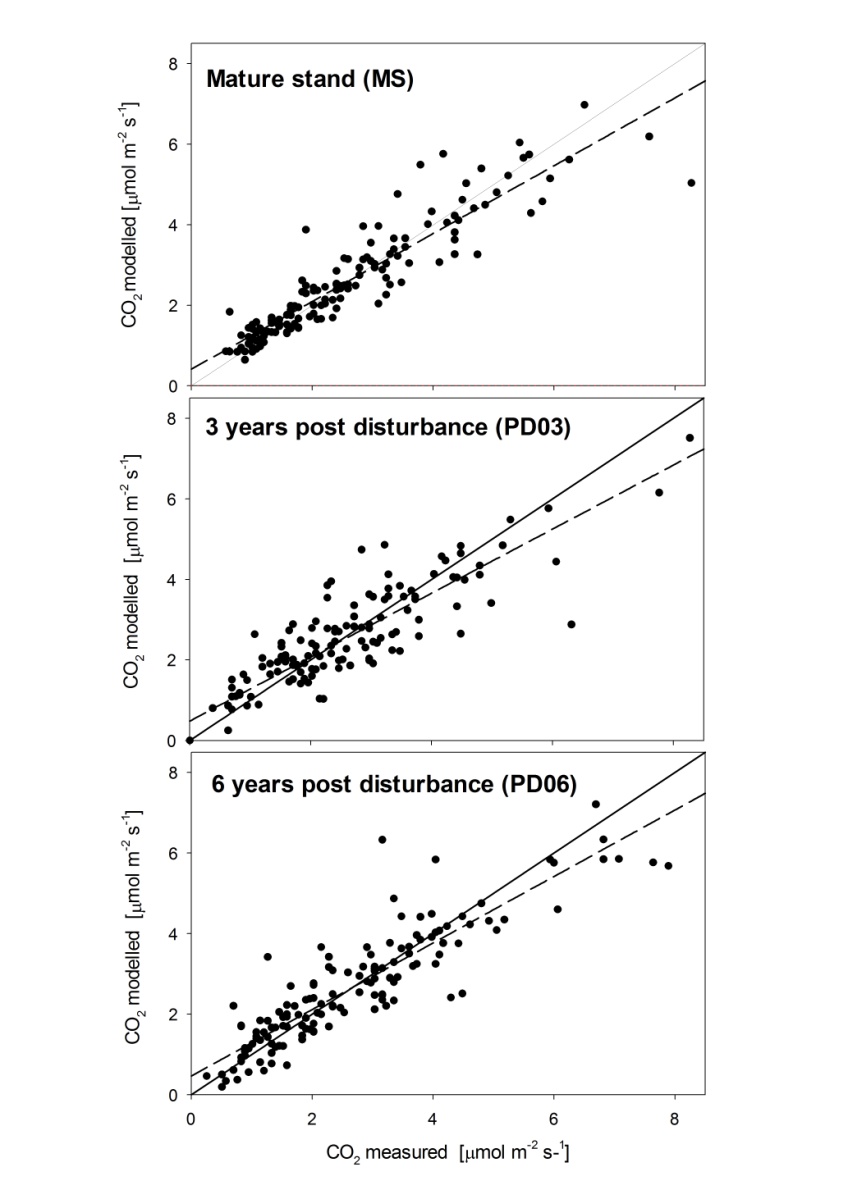


Figure A2: Measured vs. modelled soil CO_2_ efflux. Upper panel: MS (Mature stand); mid panel: PD06 (2008 Clearing); lower panel: PD03 (2011 Clearing). The linear relationship is shown by the dashed regression line.


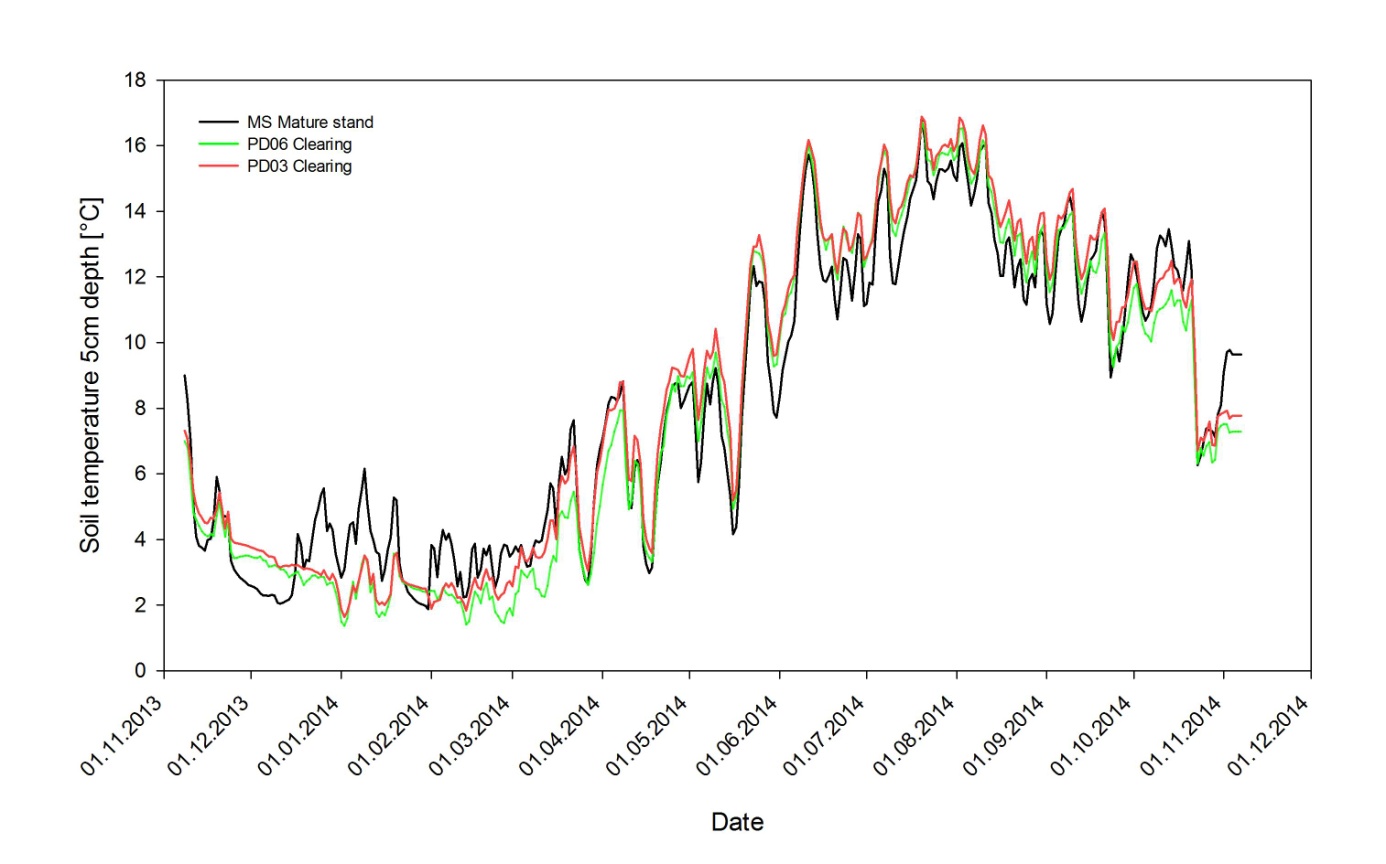


Figure A3: High resolution soil temperatures of the different stand-sections from November 2013 to November 2014.
